# A study on the association of diabetic dermopathy with nephropathy and retinopathy in patients with type 2 diabetes mellitus

**DOI:** 10.15171/jnp.2016.26

**Published:** 2016-07-26

**Authors:** Mahmoud Mirhoseini, Nasrin Saleh, Ali Momeni, Fatemeh Deris, Majid Asadi-Samani

**Affiliations:** ^1^Medical Plants Research Center, Shahrekord University of Medical Sciences, Shahrekord, Iran; ^2^Students Research Committee, Shahrekord University of Medical Sciences, Shahrekord, Iran; ^3^Cellular and Molecular Research Center, Shahrekord University of Medical Sciences, Shahrekord, Iran; ^4^Social Health Determinants Research Center, Shahrekord University of Medical Sciences, Shahrekord, Iran

**Keywords:** Type 2 diabetes, Dermopathy, Retinopathy, Nephropathy

## Abstract

**Background:**

Diabetic dermopathy is one of the most prevalent skin complications in diabetes patients. Some studies have pointed to association of diabetic dermopathy with retinopathy and nephropathy in patients with type 2 diabetes as microangiopathy presentations, but no rigorous study has been conducted to confirm this association.

**Objectives:**

This study investigated association of diabetic dermopathy with nephropathy and retinopathy in patients with type 2 diabetes referring specialty clinic of Shahrekord.

**Patients and Methods:**

This descriptive, cross-sectional study was conducted on 102 type 2 diabetes patients with dermopathy referring clinic constantly or as outpatient. Dermatological and ophthalmological examinations and examination for nephropathy were done for all patients. Demographic data and results of examinations and patients history, and biochemical tests were gathered and recorded by researcher developed checklists.

**Results:**

Mean age of patients was 83.8 2.60 years, of whom 64 (63.7%) were female and 37.3% were male. Prevalence of retinopathy in patients was 4.31% and nephropathy 3.33%. In this study, significant associations of diabetic dermopathy with diabetic nephropathy (*P* = 0.001), with retinopathy (*P* < 0.001), age (*P* < 0.001), duration of diabetes (P = 0.001), and also with glycosylated hemoglobin (*P* < 0.01) was detected. No significant association between diabetic dermopathy and other studied variables was seen (*P* > 0.05).

**Conclusions:**

Results of this study confirm the association of diabetic dermopathy with retinopathy and nephropathy in patients with type 2 diabetes. Since dermopathy is usually developed before retinopathy and nephropathy, dermopathy could be used as a clinical finding in early diagnosis and prevention of retinopathy and nephropathy in diabetes patients.

**Implication for health policy/practice/research/medical education:**

Diabetic dermopathy is prevalent and generally takes place before retinopathy and nephropathy. Few studies have so far investigated the association between systemic complications of diabetes such as retinopathy and nephropathy and obtained inconsistent findings. Since diabetic dermopathy takes place before retinopathy and nephropathy if the association between diabetic dermopathy with retinopathy and nephropathy in diabetic patients is confirmed, early diagnosis of dermopathy could be to prevent retinopathy and nephropathy. This study confirms the association of diabetic dermopathy with retinopathy and nephropathy and suggests dermopathy as a usable clinical finding in early diagnosis of retinopathy and nephropathy in patients with type 2 diabetes.

## 1. Background

The chronic complications of diabetes are divided into two types, vascular and non-vascular. The vascular complications could be microvascular (retinopathy, neuropathy, and nephropathy) and macrovascular (coronary artery disease, peripheral artery disease, and cerebrovascular disease). Non vascular complications include infection and skin and joint lesions, as well. Many of the patients with type 2 diabetes have some of these complications at diagnosis because type 2 diabetes is characterized by a long, asymptomatic period. As hyperglycemia persists, chronic complications of diabetes frequently observed since the second decade of hyperglycemia increase. Microvascular complications of type 1 and type 2 diabetes are usually developed because of chronic hyperglycemia and therefore prevention of chronic hyperglycemia helps to prevent and/or delay the incidence of retinopathy, neuropathy, and nephropathy. The role of hyperglycemia in development of macrovascular is less obvious but dyslipidemia and hypertension contribute significantly to incidence of macrovascular complications (1-4).

Diabetic retinopathy as a chronic complication of diabetes plays a significant role in declining the quality of life in diabetes patients and is an important reason for blindness in the active populations of the West (5). Retinopathy does not take place in the youth population with type 1 diabetes for at least 5-10 years after the onset of diabetes. However the patients with type 2 diabetes may have retinopathy at diagnosis (6). In a review article analyzing the findings of the studies conducted in 33 countries worldwide, the prevalence of diabetic retinopathy in the patients already diagnosed with hepatitis was reported 9.27% and in those recently diagnosed with diabetes 6.16% (7). The diagnosis and treatment of the resulted changes before decrease in vision could prevent the blindness due to diabetes (8). Another macrovascular complication of chronic diabetes is diabetic nephropathy which is the main reason for end stage renal disease in 30% of the patients with type 1 diabetes and 20% of those with type 2 diabetes. Approximately 40%-45% of the diabetes patients clearly present with the symptoms of diabetic nephropathy in lifetime (9,10). Out of the microvascular complications, diabetic dermopathy is the most prevalent skin presentation (11,12), including hyperpigmented and atrophic macules or papules with no sharp border and usually occurring on the front leg in diabetes patients. These lesions are asymptomatic and often do not cause pain and itching. The diagnosis of diabetic dermopathy is clinical and is made by description and physical examination. The occurrence of scars and hyperpigmented, atrophic spots with sharp borders on the legs of a diabetes patients is very likely to result in diagnosis with diabetic dermopathy (11,13,14). Diabetic dermopathy has been reported 9%-55% prevalent and is likely to be more prevalent in the patients over 50 years and with longer duration of diabetes. Meanwhile, it occurs usually before diabetic retinopathy and nephropathy (12,15). Genetic predisposition could contribute to developing most of the complications because retinopathy or neuropathy does not occur in some of the patients with prolonged duration of diabetes. However the mortality from heart coronary disease is two to four times higher in the patients with type 2 diabetes. These events are associated with fasting blood sugar, non-fasting blood sugar, and also HbA1c (16-18) and hence all the factors involved in developing sensitive complications should be studied.

Several studies have examined the association between retinopathy and nephropathy in diabetes patients, most of which have considered the incidence of retinopathy as necessary for nephropathy diagnosis (9,19,20). However few studies have so far investigated the association between diabetic dermopathy and certain predisposing demographic characteristics and also other systemic complications of diabetes such as retinopathy and nephropathy and obtained inconsistent findings. Some of them reported an association and some others found no or statistically insignificant association (14,17,21-24).‏ Therefore since diabetic dermopathy is prevalent and generally takes place before retinopathy and nephropathy, early diagnosis of dermopathy could help to predict the incidence of retinopathy and nephropathy, delay their incidence and adverse and dangerous consequences, or slow down their progression by managing diabetes and other factors more efficiently if the association between dermopathy and incidence of retinopathy and nephropathy is confirmed in the patients with type 2 diabetes.

## 2. Objectives

This study aimed to more closely examine and probably confirm the association between dermopathy and incidence of retinopathy and nephropathy in the patients with type 2 diabetes and also to investigate some dermopathy-associated demographic characteristics.

## 3. Patients and Methods

This descriptive-analytical study was conducted on all diabetes patients admitted to Imam Ali clinic of Shahrekord from 2012 to 2014. The inclusion criteria into the study were suffering from type 2 diabetes and incidence of skin lesions characterized as diabetic dermopathy. Other diabetes patients and the patients unwilling to participate in the study were excluded. The sample size was determined as 102 individuals with reference to a previous study (23) and sample size formula.

For this study, 102 patients with type 2 diabetes and diagnosed with dermopathy by clinical examination were enrolled. These patients were examined for demographic characteristics, hypertension, and diabetic dermopathy lesions in both legs and the data were recorded in a researcher-developed checklist.

The patients were referred to an ophthalmologist to be examined for retinopathy and its type. Also 24-hour urine test was asked from the patients to measure their proteinuria, urine volume, and creatinine for nephropathy diagnosis and determination of its severity. The patients with nephropathy were divided into two groups based on 24-hour urine protein amount, mild nephropathy and severe. In addition, fasting blood sugar (FBS) and HbA1c were measured.

### 3.1. Ethical issues

1) The research followed the tenets of the Declaration of Helsinki; 2) informed consent was obtained, and they were free to leave the study at any time; and 3) the research was approved by the ethical committee of Shahrekord University of Medical Sciences.

### 3.2. Statistical analysis

The data were encoded and entered into SPSS 18. For data analysis, descriptive and analytical (independent *t* test, chi-square, Mann-Whitney U test, and Pearson’s correlation coefficient) statistical tests were used and *P* value less than 0.05 was considered as significant.

## 4. Results

Of 102 patients in this study, 64 (62.7%) were women. The patients aged 38-80 years old with mean age of 60.2±8.38 years. Of the patients, 63.7% had hypertension and 71 (69.6%) had family history of diabetes. Mean duration of diabetes was reported 9.16±5.24 (range: 1-30) years. Most (62.7%) patients used oral medications to control diabetes and the rest took insulin. Mean level of FBS and HbA1c was 123.14±48.87 mg/dL and 7.29±1.24 % respectively.

In this study the mean proportion of dermopathy lesions was 9.7±5.34. Of 34 (33.3%) patients with nephropathy, 21 had mild dermopathy and the rest had severe dermopathy. Retinopathy was diagnosed in 32 (31.4%) patients, 25 of whom had nonproliferative retinopathy and only 7 had proliferative retinopathy.

Independent *t* test was used to compare the mean proportion of lesions in the patients with concurrent dermopathy and retinopathy and those with dermopathy alone, and confirmed a significant association between dermopathy and retinopathy (*P*<0.001). The association between mean proportion of lesions in the patients with concurrent dermopathy and nephropathy and those with dermopathy alone was examined by independent t-test and a significant association between dermopathy and nephropathy was seen (*P*<0.001). Furthermore, a significant association between insulin injection and proportion of dermopathy lesions was detected (*P*<0.05). Likewise, the proportion of lesions in the patients with dermopathy was not significantly associated with gender and hypertension history (*P*>0.05; Table 1).

**Table 1:** The association between proportion of dermopathy lesions and the variables under study by independent *t* test

**Variables**	**SD±Mean**	**Proportion of patients**	**P**
Retinopathy	4.85±13.16	32	<0.001
Nephropathy	5.45±12.15	34	0.001
Gender^a^	5.56±9.75	64	0.896
Hypertension history	3.03±5.15	65	0.055
Insulin intake	2.93±4.40	64	0.030

^a^For female participants.

Pearson’s correlation coefficient was used to examine the association between the number of dermopathy lesions and body mass index (BMI), duration of diabetes, age, and FBS and HbA1c levels and confirmed a significant association between the number of these lesions and duration of diabetes, age and HbA1c level (Table 2). Mann-Whitney test indicated that the number of dermopathy lesions was not significantly associated with nephropathy severity (*P*>0.05).

**Table 2:** The association between proportion of dermopathy lesions and other variables under study by Pearson’s correlation coefficient

**Variables**	**Pearson's correlation**	**P**
Body mass index	0.019	0.851
Duration of diabetes	0.312	0.001
Age	0.417	<0.001
Fasting blood sugar	0.174	0.080
HbA1c	0.262	0.008

## 5. Discussion

The present study was conducted to more precisely study and probably confirm an association between dermopathy and incidence of retinopathy and nephropathy in the patients with type 2 diabetes as well as to examine some dermopathy-associated demographic characteristics. The findings demonstrated a significant association of dermopathy with retinopathy, nephropathy, age, duration of diabetes, and HbA1c.

In this study, the mean age of the patients was 60.2±8.83 years and there was a significant association between incidence of diabetic dermopathy and age. In other studies, diabetic dermopathy has been reported most prevalent in 45- to 70-year-old patients and significantly associated with age (11,14,15,25), as well. Further, the mean duration of diabetes was 9.16±5.42 years and the number of dermopathy lesions was significantly associated with the duration of illness, which is in agreement with other studies (17,21,26). This consistency of the findings further confirms the association between diabetes duration and the number of dermopathy lesions. In addition, mean HbA1c level in the present study was 7.29±1.24 which was significantly associated with the number of dermopathy lesions. This finding confirms other studies’ findings on the association of HbA1c with diabetic dermopathy (17,26). Another finding in this study was the significant association between diabetic dermopathy and insulin intake, which is in line with a study reporting a significantly lower incidence of dermopathy in the patients under treatment with insulin than those taking oral medications (21).

Of 102 type 2 diabetes patients with dermopathy studied in the present study, 32 (31.4%) had diabetic retinopathy, seven of whom had proliferative retinopathy and the rest nonproliferative retinopathy. And a significant association was seen between dermopathy and retinopathy. In a study on 25 type 1 diabetes patients with dermopathy, approximately half (12, 48%) of the patients had retinopathy (27). Also in Abdollahi et al study, 30 (44%) patients had concurrent dermopathy and retinopathy with a significant association between them (24), which is consistent with the present study. Another complication examined in the present study was nephropathy whose association with diabetic dermopathy was studied and a significant association between diabetic dermopathy and nephropathy was seen in the patients with type 2 diabetes. Shemer et al study found a significant association between dermopathy and nephropathy (21), as well. Moreover Brugler et al study reported that 72% of type 1 diabetes patients with dermopathy suffered from nephropathy simultaneously (27).

According to the findings of the present study, 64 patients with dermopathy were men and 38 were women and no significant association between gender and proportion of lesions due to diabetic dermopathy was seen. According to the available references, the prevalence of diabetic dermopathy is higher in elderly men (28), and some other studies have demonstrated that diabetic dermopathy is more prevalent in men with a significant association between gender and diabetic dermopathy (11,22,24). These findings are consistent with our investigation, which could be explained by lower number of the men than women referring to our clinic. Furthermore, in this study no significant association between high BMI and proportion of dermopathy lesions was seen. This finding was in agreement with a study of, which considered the association between skin lesions, microalbuminoria, and other macrovascular complications in the patients with type 2 diabetes in 2010. This study showed BMI was not associated with dermopathy and other studied factors (23).

## 6. Conclusions

In the present study, a significant association between dermopathy and age, duration of diabetes, retinopathy, and nephropathy was seen. Therefore as the incidence of diabetic retinopathy and nephropathy is the same in the patients with diabetes, it is recommended that patients with diabetic dermopathy be examined for retinopathy and nephropathy as soon as possible so that, these complications could be prevented or controlled more efficiently.

## Limitations of the study

Low proportion of male patients with type 2 diabetes referred to the clinic was the limitation of this study.

## Acknowledgment

This study is adapted from the M.D. thesis of Nasrin Saleh. We gratefully thank the Research and Technology Deputy of this University and all the patients and people who assisted us in conducting this thesis.

## Conflicts of interest

The authors declared no competing interests.

## Authors’ contribution

NS and AM, and MM collected the data and MM recruited and followed-up the patients. MA and FD performed the statistical analysis. NS and MA wrote the paper. MM and AM edited the final draft. All authors signed the manuscript.

## Funding/Support

This study was a funded thesis at Shahrekord University of Medical Sciences (Grant# 1000).
